# Supplementary material for: Benchmarking and Validation of a Bioinformatics Workflow for Meat Species Identification Using 16S rDNA Metabarcoding
Source: Foods. 2023 Feb 24;12(5):968. doi: 10.3390/foods12050968 (PMC10000984; doi:10.3390/foods12050968)
Supplement: Supplementary file 1 [file foods-12-00968-s001.zip › foods-2121010-supplementary/Figure S2.pptx]

## Slide 1
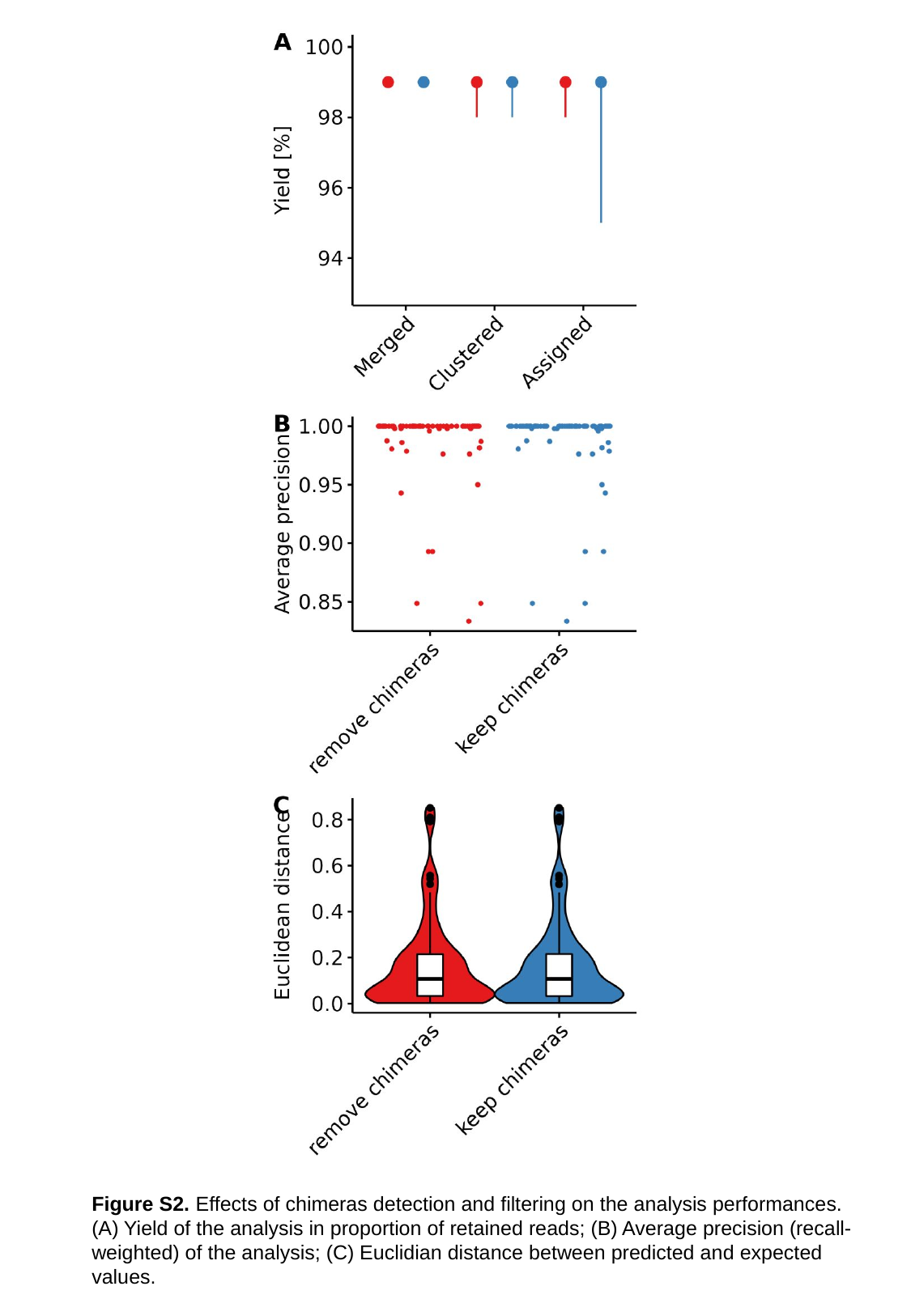

Figure S2. Effects of chimeras detection and filtering on the analysis performances. (A) Yield of the analysis in proportion of retained reads; (B) Average precision (recall-weighted) of the analysis; (C) Euclidian distance between predicted and expected values.
