# Supplementary material for: Benchmarking and Validation of a Bioinformatics Workflow for Meat Species Identification Using 16S rDNA Metabarcoding
Source: Foods. 2023 Feb 24;12(5):968. doi: 10.3390/foods12050968 (PMC10000984; doi:10.3390/foods12050968)
Supplement: Supplementary file 1 [file foods-12-00968-s001.zip › foods-2121010-supplementary/Figure S4.pptx]

## Slide 1
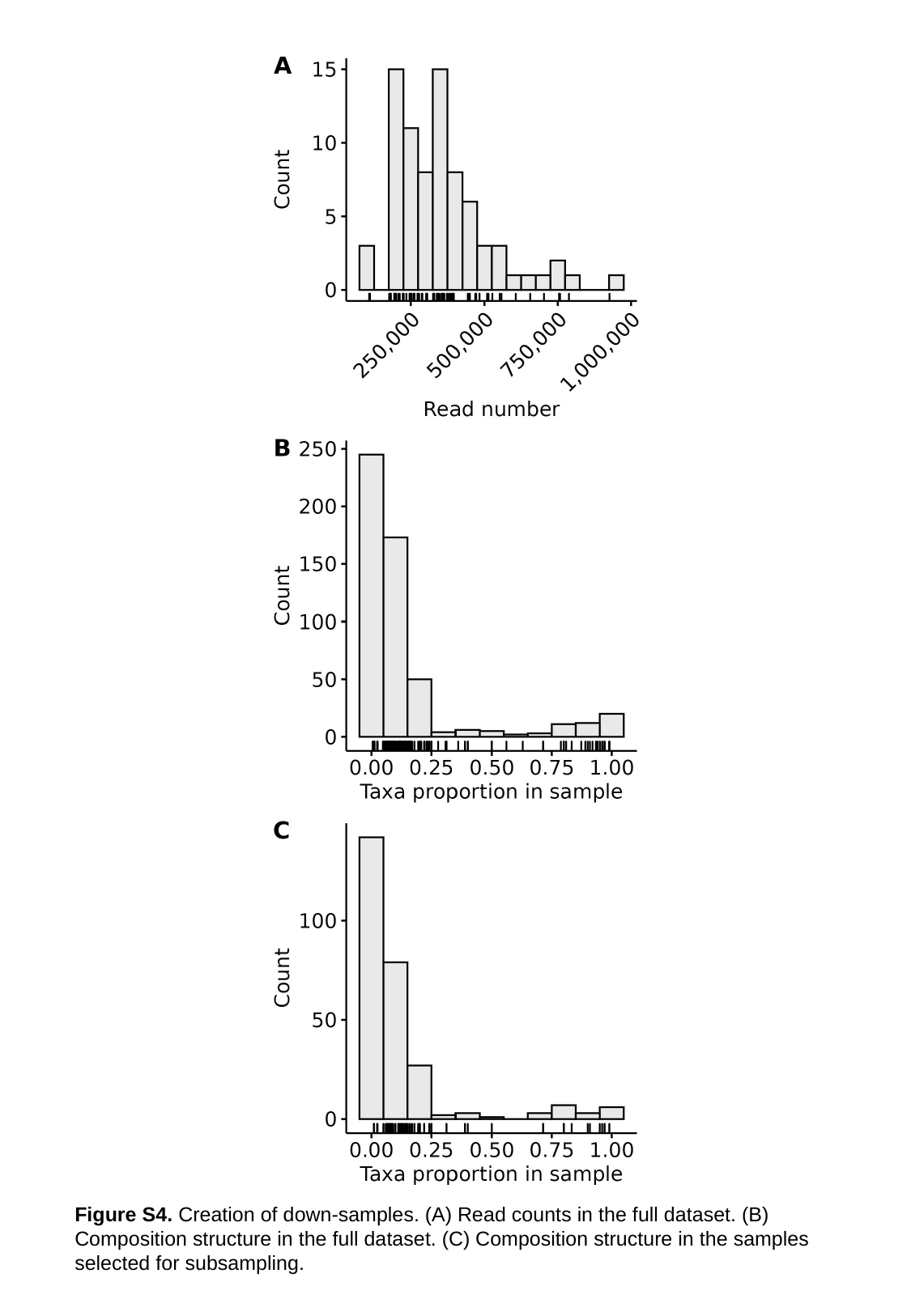

Figure S4. Creation of down-samples. (A) Read counts in the full dataset. (B) Composition structure in the full dataset. (C) Composition structure in the samples selected for subsampling.
